# Non-Accidental Injuries in Pediatric Primary Care: A Systematic Literature Review of Objective Assessment Tools and Trends in Gulf Cooperation Council Region

**DOI:** 10.7759/cureus.35819

**Published:** 2023-03-06

**Authors:** Muhannad Q Alqirnas, Faisal Alrasheed, Mohammed I Alhumaidan, Mansour Alrasheed, Yasser Alkharashi, Abdulaziz S Almosa, Eyad K Althaqeb, Shamayel A Almulhem, Sadeem A Khallaf, Rayan S Almahmoud, Bader AlTulaihi

**Affiliations:** 1 College of Medicine, King Saud Bin Abdulaziz University for Health Sciences, King Abdullah International Medical Research Center, Riyadh, SAU; 2 Department of Family Medicine, Ministry of National Guard-Health Affairs, King Abdullah International Medical Research Center, Riyadh, SAU; 3 College of Medicine, Imam Mohammad Ibn Saud Islamic University, Riyadh, SAU; 4 College of Medicine, Alfaisal University, Riyadh, SAU

**Keywords:** gcc, gulf cooperation council, child safety, non-accidental trauma, child abuse and neglect, pediatric fractures

## Abstract

Nonaccidental injuries (NAI) in pediatric primary care in the Gulf Cooperation Council (GCC) region refer to intentional injuries inflicted on children, usually by a caregiver or a family member. The region has seen an increase in nonaccidental injuries in recent years, and healthcare providers have a crucial role in identifying and managing the cases. The aim of research on nonaccidental injuries in pediatric primary care in the GCC region is to identify objective assessment tools and evaluate trends in the region.

PubMed, Google Scholar, and EMBASE databases were searched using search string keywords. The search was conducted on 7^th^ February 2023. The keywords included in the search included the search terms: nonaccidental injuries, pediatric, and GCC. All the studies were published between 1990 and 2023. The articles that passed the eligibility criteria were read fully to examine whether they would be relevant to the current systematic literature review.

An initial search identified 3059 studies (Google Scholar = 6, EMBASE =12, PubMed = 4732). After the deletion of similar articles, only 3613 articles were left for further screening. The abstracts and titles of the left articles were scanned and screened to decide whether they were included in this systematic review. After deletion, 16 papers were inspected and read fully based on predefined eligibility criteria. Eventually, 11 papers were recognized and identified that passed the eligibility criteria of this systematic review.

The studies reviewed in this analysis highlight the significant problem of child abuse in the GCC region, particularly neglect as the most common form of abuse. The studies also suggest that parents are the most frequent perpetrators of abuse. The need for policies and interventions to prevent child abuse and support victims is paramount.

## Introduction and background

**Figure 1:**
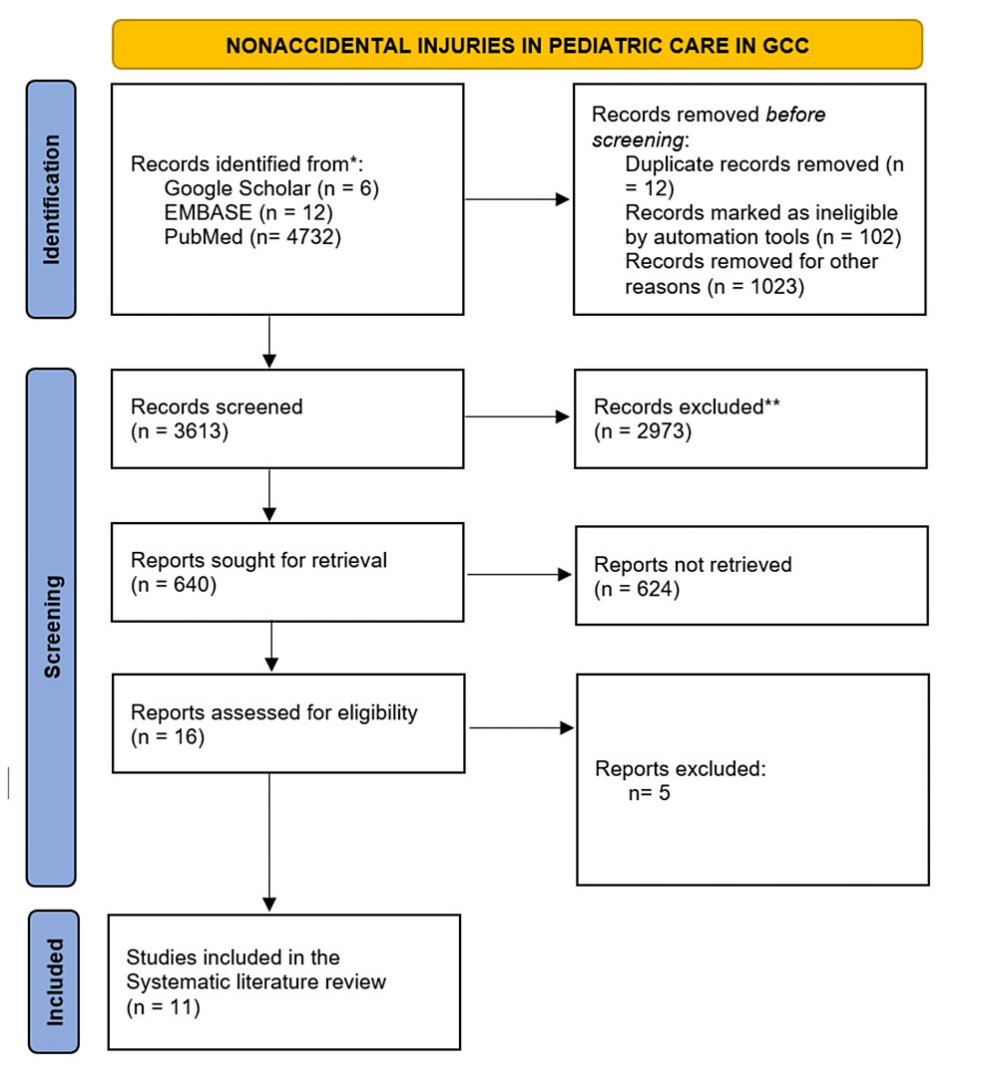
Prisma Flow Diagram of Inclusion/Exclusion Criteria

The issue of nonaccidental injuries (NAI) in pediatric primary care is a great concern in the Gulf Cooperation Council (GCC) region. Child abuse, a pervasive global problem, has been documented by the World Health Organization's Global Status Report, which indicates that physical abuse during childhood was experienced by around one-fourth of adults from most countries [1]. Approximately 300 million children, or nearly three in four children between the ages of two and four, are subject to recurrent physical punishment or psychological violence from their parent or caregiver figures [1]. One in every five women and one in 13 men report a history of sexual abuse during childhood, ranging from birth to age 17 [1]. Additionally, 120 million girls and young women under the age of 20 have been subjected to forced sexual contact [1]. The long-term consequences of child maltreatment include poor physical and mental health throughout the victim's lifetime and adverse social and occupational outcomes that could decelerate a nation's economic and social development. Child maltreatment frequently remains concealed, with only a fraction of the afflicted individuals receiving aid from healthcare professionals [2]. Furthermore, a child who has experienced maltreatment is more prone to inflict similar harm on others as an adult, thus perpetuating the cycle of violence through future generations [3]. Therefore, it is critical to disrupt this cycle of violence and foster a positive, multi-generational impact by preventing child maltreatment before it commences through a comprehensive, cross-sectoral approach.

Prompt recognition would expedite child protection care and prevent further harm [3]. However, physicians often misdiagnose the physical abuse of children because the caregivers intentionally give a fake history to mislead the physicians. For instance, a study conducted in Canada found that in pediatrics under the age of three years, the percentage of misdiagnosed or misclassified fractures caused by abuse is as high as 20% [4].

Nonaccidental fractures (NAF) come second among the most common accidents among physically abused pediatrics; approximately one-third of physically abused children present with skeletal fractures [4]. Orthopedic surgeons play an important role in evaluating and detecting potentially abused children, as one of the most common presentations of such cases is bone fractures. Thus, it is essential for all healthcare workers, especially orthopedic surgeons, to be familiar with the features of fractures resulting from physical abuse, as many children present with isolated fractures. The most commonly reported musculoskeletal manifestations of child abuse cases include humeral, skull, rib, and femoral fractures, and radiographic imaging is often essential to accurately diagnose child abuse [4]. Objective assessment tools play a vital role in accurately diagnosing child abuse cases. It is imperative that healthcare professionals involved in the diagnosis and treatment of pediatric patients be knowledgeable about the clinical characteristics and diagnostic methods for identifying physical abuse in children. This systematic review aims to identify the assessment tools and trends associated with nonaccidental injuries in the Gulf Cooperation Council region.

## Review

Inclusion criteria

The studies were selected for the systematic literature review depending on whether they met the eligibility criteria. All the articles had to have been published between 1990 and 2023. The studies needed to include children participants below the age of 16 exclusively. All the articles had to have been published in the English language and published through the correct channels. The studies had to focus specifically on nonaccidental injuries limited to children in the GCC region. The full-text sources had to be available online. The studies also had to report the number of patients and evidence of nonaccidental injuries. The study designs allowed for the systematic literature review case series, case reports, and prospective and retrospective studies. Considering the nature of the research topic, clinical trials such as randomized controlled trials and comparative studies could not be included since the outcomes are post-injury and, therefore, cannot be controlled.

Exclusion criteria

Unpublished papers, books, newspapers, dissertations, literature reviews, narrative papers, and theses were excluded. Grey literature was also excluded during the selection process. The studies where the age limit for the participants was greater than 16 years were also excluded. The analysis did not include studies where the injuries were accidental or caused by an illness. Reviews were also excluded since they could affect the integrity of the results. Sources with only available abstracts were also excluded; only full-text online sources were included.

Search strategy

PubMed, Google Scholar, and EMBASE databases were searched using search string keywords. The search was conducted on 7^th^ February 2023. The keywords included in the search included the search terms: nonaccidental, injuries, pediatric, and GCC. The keywords were combined to optimize the results. All the studies were published between 1990 and 2023. The data runs for each database were conducted individually, and the data from both were combined and scrutinized.

Keywords

The following keywords were used: Nonaccidental injuries, pediatric care, GCC, child abuse, pediatrics, abusive head trauma, physical abuse, neglect, traumatic brain injury, shaken baby syndrome. For more data regarding the key wording, strings, and searching techniques refer to Table 1.

**Table 1:** Searching Strings That Are Used for the Screening of Articles GCC = Gulf Cooperation Council

Number	Search String
#1	(("Nonaccidental injuries" OR "Child abuse" OR "Abusive head trauma" OR "Physical abuse" OR "Neglect" OR "Nonaccidental trauma" OR "Shaken baby syndrome" OR "Abusive burns" OR "Traumatic brain injury" OR "Munchausen syndrome by proxy") AND ("Pediatric care" OR "Pediatrics") AND "GCC")
#2	("Nonaccidental injuries" OR "Child abuse" OR "Abusive head trauma" OR "Physical abuse" OR "Neglect" OR "Nonaccidental trauma" OR "Shaken baby syndrome" OR "Abusive burns" OR "Traumatic brain injury" OR "Munchausen syndrome by proxy") AND ("Pediatric care" OR "Pediatrics") AND ("Gulf Cooperation Council" OR "GCC")
#3	("Nonaccidental injuries" OR "Child abuse" OR "Abusive head trauma" OR "Physical abuse" OR "Neglect" OR "Nonaccidental trauma" OR "Shaken baby syndrome" OR "Abusive burns" OR "Traumatic brain injury" OR "Munchausen syndrome by proxy") AND ("Pediatric care" OR "Pediatrics") AND ("Bahrain" OR "Kuwait" OR "Oman" OR "Qatar" OR "Saudi Arabia" OR "United Arab Emirates")
#4	("Nonaccidental injuries" OR "Child abuse" OR "Abusive head trauma" OR "Physical abuse" OR "Neglect" OR "Nonaccidental trauma" OR "Shaken baby syndrome" OR "Abusive burns" OR "Traumatic brain injury" OR "Munchausen syndrome by proxy") AND ("Pediatric care" OR "Pediatrics") AND ("Persian Gulf" OR "Arabian Gulf")
#5	("Nonaccidental injuries" OR "Child abuse" OR "Abusive head trauma" OR "Physical abuse" OR "Neglect" OR "Nonaccidental trauma" OR "Shaken baby syndrome" OR "Abusive burns" OR "Traumatic brain injury" OR "Munchausen syndrome by proxy") AND ("Pediatric care" OR "Pediatrics") AND ("Middle East" OR "Arabian Peninsula")
#6	("Nonaccidental injuries" OR "Child abuse" OR "Abusive head trauma" OR "Physical abuse" OR "Neglect" OR "Nonaccidental trauma" OR "Shaken baby syndrome" OR "Abusive burns" OR "Traumatic brain injury" OR "Munchausen syndrome by proxy") AND ("Pediatric care" OR "Pediatrics") AND ("Healthcare" OR "Medical care" OR "Clinical practice")

The sources were combined, downloaded, and imported into the Zotero application, which is an open-source application that helps in managing bibliographic data that are research related. The articles were checked for duplication, and the duplicates were eliminated. The titles of the articles were checked to assess their relevance to the research topic. The abstracts of the selected articles and the remaining full-text sources were assessed to determine whether they met the inclusion criteria. The articles that failed to meet the inclusion criteria were eliminated. The articles that passed the eligibility criteria were read fully to examine whether they would be relevant to the current systematic literature review. The final articles were included in the systematic literature review. References of the included articles were also checked for other potential sources for the review.

Study selection

An initial search identified 3059 studies (Google Scholar = 6, EMBASE =12, PubMed = 4732). After the deletion of the identical articles, only 3613 studies were left for further screening. The abstracts and titles of the 3613 studies were scanned and screened to determine their significance for this systematic review. After the screening of the 3613 studies, 3597 articles were excluded due to not meeting the eligibility criteria. Sixteen articles were further screened utilizing their full text; they were scrutinized and inspected based on predefined eligibility criteria. Finally, 11 articles were identified that effectively passed the eligibility criteria(Figure 1).

The data extraction table provides information on studies related to child abuse in the GCC (Gulf Cooperation Council) region, including Saudi Arabia, Kuwait, Bahrain, and the UAE. The studies range from retrospective to cross-sectional and case reports, and most involve a small number of participants (Table 2).

**Table 2:** Data Extraction Table of the Included Studies UAE = United Arab Emirates

Author	Year	Study Design	Participants	GCC Region	Evidence of Nonaccidental Injury	Results
Jawadi et al. [4]	2019	Retrospective Study	512 children (aged ≤14 years) registered in the National Family Safety Program Registry	Riyadh, Saudi Arabia	103 fractures were identified. Neglect is the most common cause of nonaccidental fractures, abusive head trauma is the most commonly associated injury, and transverse fracture is the primary pattern of fracture in abused children [4].	Nearly 79% of children presented with a single bone fracture, while 21% had multiple bone fractures. The most common sites of fractures were the skull (40%), upper limbs (31%), and lower limbs (29%). The most common fracture pattern was transverse fractures (48%), and it was mainly diagnosed in skull fractures (51%) [4].
Bener, A., el-Rufaie, O. E., & al-Suweidi [5]	1997	Retrospective Study	6,518 (69.9% boys; 30.1% girls) patients aged 0-14 years	Al-Ain, United Arab Emirates (UAE)	The most common causes were falls, blunt trauma, and burns or scalds [5].	The majority of cases occurred among non-UAE nationals, who are usually of lower socio-economic status [5].
Maha et al. [6]	2022	Retrospective Study	309 patents (Mean age of victims was 4.4±4.1 years, and 51.8% were male gender)	Riyadh, Saudi Arabia	Neglected children have a higher incidence of internal injuries, superficial injuries, burns, ingestion, and poisoning compared to non-neglected children [6],	Worsening of the underlying disease (30%) and internal injuries (23.5%) were the most common consequences of neglect. Mortality attributed to neglect was documented in eight (2.6%) children [6].
Al‐Eissa et al. [7]	2016	Cross-sectional study	(n = 16 939) of adolescents (15-19 years)	Saudi Arabia	Greater rates of all forms of abuse/exposure were found when participants lived with their mother or father only (versus with both) [7].	Rates for violence exposure, psychological abuse, and neglect were significantly greater for girls, and the rate of sexual abuse was greater for boys [7].
Al-Ateeqi, W., Shabani, I., & Abdulmalik, A. [8]	2002	Retrospective Study	60,640 records, 16 children showed evidence of abuse	Kuwait	Bruises (77%), burns (38%), intracranial hemorrhage (38%), fractures (23%), and cut wounds (15%) [8].	The perpetrator was a parent in 75% of the cases, which involved the following abuses: physical, 13; sexual, 2; and Munchausen syndrome by proxy in 1 [8].
Kattan H. [9]	1994	Retrospective study	10 Patients	Saudi Arabia	Injuries included bruises, fractures, bleeding, and intracranial hemorrhage [9].	Four were physically abused, two were sexually abused, and four suffered from MSP [9].
Al Ayed, I. H., Qureshi, M. I., Al Jarallah, A., & Al Saad, S. [10]	1998	Retrospective study	13 children	Riyadh, Saudi Arabia	There were four cases of nonaccidental injury, three of which had serious injuries [10].	There were three cases of sexual abuse, and four cases of neglect, resulting in the death of one child and severe emaciation in another. There was one suspected case of Munchausen syndrome by proxy [10].
Elkerdany, A. A., Al-Eid, W. M., Buhaliqa, A. A., & Al-Momani [11]	1999	Case report	2 children	Saudi Arabia	Extensive intracranial injuries, bilateral subconjunctival hemorrhage and hyphema bilateral vitreous hemorrhage, and several fractures, bleeding from the left eye [11].	Policies should be implemented to prevent the reoccurrence of such nonaccidental injuries [11].
Roy, D., Al Saleem, B. M., Al Ibrahim, A., & Al Hazmi [12]	1999	Case report	1 Child	Saudi Arabia	Rhabdomyolysis, acute renal failure, ecchymoses, scars, swollen and tender thighs and buttocks [12].	
Karthikeyan, G., Mohanty, S. K., & Fouzi [13]	2000	Case report	3 Children	Saudi Arabia	Injuries included fractures, bruises, bite marks, bilateral retinal hemorrhage, cerebral contusion, subdural hematoma, cerebral infarction, and one child died. Injuries in sexual abuse included anal tears and painful anal introitus [13].	Children received medical, orthopedic, and supportive care, but none received social or psychiatric care or police referral [13].
Al-Mahroos, F., Abdulla, F., Kamal, S., & Al-Ansari [14]	2005	Case series	184 girls	Bahrain	The most common manifestations of physical abuse were bruises in 31 (45%) and burns in 15 (27%). Fractures were identified in 25% and head injuries in 19% of the children. Three cases (5%) had abdominal injuries (ruptured intestine, peritonitis, and duodenal hematoma). Ten (19%) of the patients were critically ill, and four patients died (two with head injuries) [14].	Males were more likely to be physically abused than girls (63% vs. 37%, respectively). The abusers were males in 52% and females in 48% of the cases. Similarly, the most common manifestations of physical abuse were bruises (45%), followed by burns (27%) and fractures (25%) [14].

Analysis

**Figure 2:**
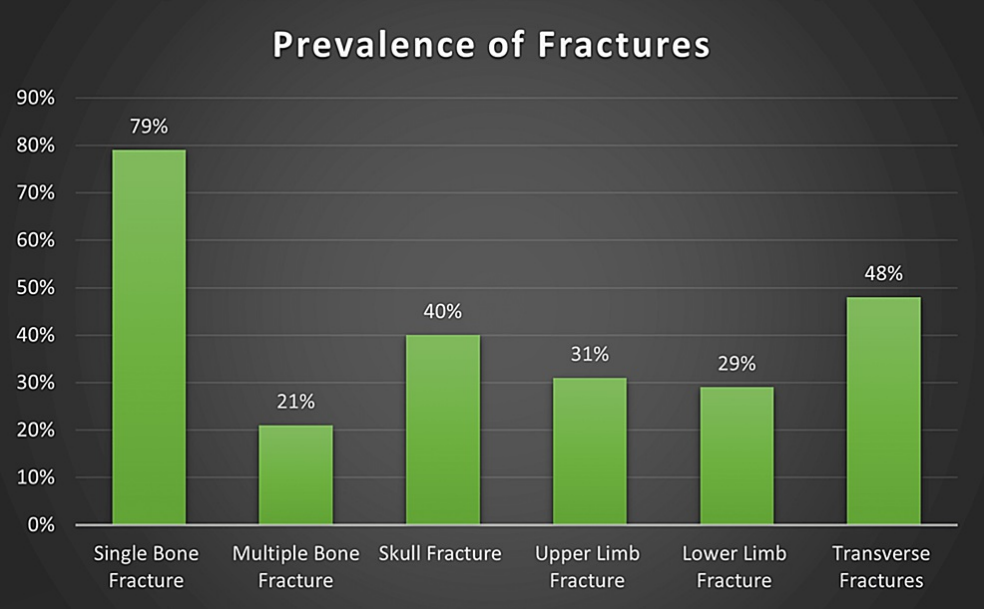
Prevalence of Fractures in Pediatric Nonaccidental Injuries in GCC

The prevalence of bone fractures in nonaccidental injuries is as follows: single bone fracture is the most common at 79%, followed by transverse fractures at 48% (Figure 2).

**Figure 3:**
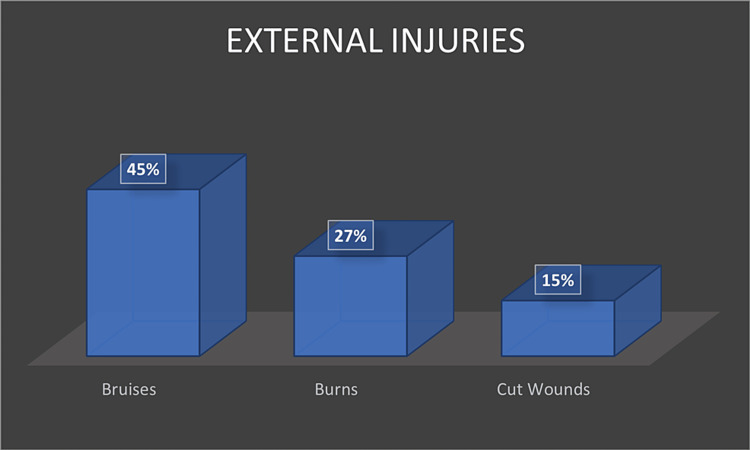
Prevalence of External Injuries

Regarding external injuries, bruises were the most common observed injury (45%), followed by burns (27%) (Figure 3).

**Figure 4:**
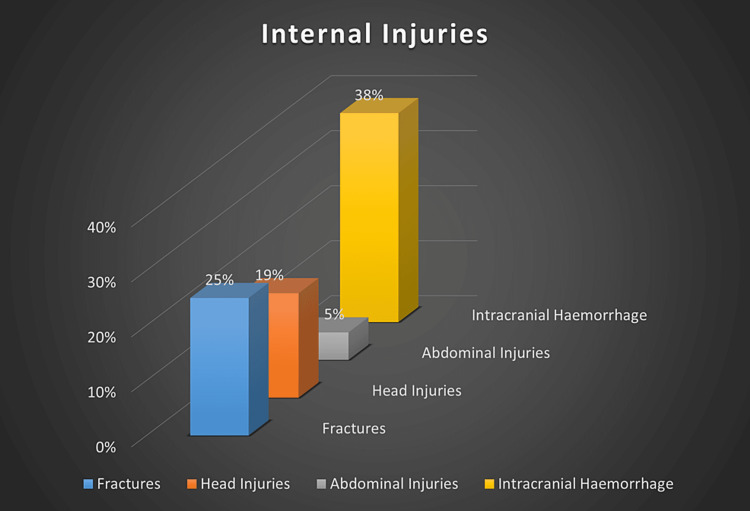
Prevalence of Internal Injuries

Regarding internal injuries, intracranial hemorrhage (38%) was the most common observed injury among our population in our included studies, followed by fractures (25%) and then head injuries (19%) (Figure 4).

Objective assessment tools

Unfortunately, most studies failed to mention the objective assessment tools used to screen nonaccidental injuries in pediatric care in GCC. The only mentioned tool was from the International Society for the Prevention of Child Abuse and Neglect (ISPCAN), which is called Child Abuse Screening Tool (ICAST). The ICAST is a screening tool that was designed to be used by professionals across different disciplines to identify children who may be at risk of abuse or neglect. It consists of a 10-item questionnaire that covers four types of abuse: physical abuse, sexual abuse, emotional abuse, and neglect. The questionnaire is designed to be administered by professionals trained in its use. In the context of nonaccidental injuries in pediatric primary care, it is a valuable tool for healthcare providers to identify cases of abuse or neglect and provide timely and appropriate interventions to prevent further harm.

Quality assessment of the included studies****

The included studies were assessed using Newcastle-Ottawa Scale (NOS) as it is the most suitable due to the nature of the included studies were mostly retrospective and case reports (Table 3).

**Table 3:** Quality Assessment of the Articles Using Newcastle-Ottawa Scale NOS = Newcastle-Ottawa Scale

Author	Year	Representativeness of the exposed cohort	Selection of the non-exposed cohort	Definition of inclusion and exclusion criteria	Similarity of groups at baseline	Control for important prognostic factors	Definition of outcome and follow-up	Comparison of outcome in exposed and non-exposed groups	Assessment of outcome independently	Total Score
Jawadi et al. [4]	2019	* *	0	*	*	0	*	*	*	7
Bener, A., el-Rufaie, O. E., & al-Suweidi [5]	1997	* *	0	*	*	0	*	*	*	7
Maha et al. [6]	2022	* *	0	*	*	0	*	*	*	7
Al‐Eissa et al. [7]	2016	* *	0	*	*	0	*	*	*	7
Al-Ateeqi, W., Shabani, I., & Abdulmalik, A. [8]	2002	* *	0	*	*	0	*	*	*	7
Kattan H. [9]	1994	* *	0	*	*	0	*	*	*	7
Al Ayed, I. H., Qureshi, M. I., Al Jarallah, [10]	1998	* *	0	*	*	0	*	*	*	7
Elkerdany, A. A., Al-Eid, W. M., Buhaliqa, A. A., & Al-Momani [11]	1999	* *	0	*	*	0	*	*	*	7
Roy, D., Al Saleem, B. M., Al Ibrahim, A., & Al Hazmi [12]	1999	* *	0	*	*	0	*	*	*	7
Karthikeyan, G., Mohanty, S. K., & Fouzi [13]	2000	* *	0	*	*	0	*	*	*	7
Al-Mahroos, F., Abdulla, F., Kamal, S., & Al-Ansari [14]	2005	**	0	*	*	0	*	*	*	7

Discussion

The mistreatment of children is the most extreme manifestation of a society's disregard for its vulnerable members and their basic human rights. In the context of the GCC, the issue of non-accidental injuries in pediatric care is a multifaceted problem compounded by cultural, social, and economic factors [15]. Despite prevailing notions that the incidence of child abuse in this region is low, the assertion lacks a foundation of empirical evidence. It thus cannot be considered a substantiated claim based on scientific research. The insufficiency of empirical evidence should not be taken as an indication of a low occurrence rate. Acknowledging this fact as an essential measure in the difficult process of addressing, mitigating, and preventing instances of child neglect and abuse is imperative.

Limited data availability makes recognizing the extent of the problem a challenging task. The most severe cases of child abuse and neglect are the ones that receive medical attention, as families are forced to seek medical advice due to the gravity of the situation. [16]. However, these likely reported cases only represent a small portion of the total cases, as less severe abuse and neglect often go unreported and unrecognized [17]. The available data indicates realities regarding child neglect abuse in the region, with cases often being ignored or even perceived as disciplinary action against the children. [18]. The attitude has dire consequences, as children who are abuse victims are left to suffer and may even face fatal outcomes. Recognizing the intricate patterns, defining characteristics, and multifaceted risk factors of child abuse and neglect is essential to comprehending and addressing this pervasive problem. It is vital to understand how different types of abuse may manifest and the underlying factors that contribute to their occurrence. We can develop more targeted and effective prevention and intervention strategies by identifying the various patterns and risk factors.

The prevalence of non-accidental injuries in pediatric care is a multifactorial issue that is shaped by a complex interplay of cultural, social, and economic factors. Cultural attitudes towards child-rearing practices, such as accepting physical punishment, contribute to normalizing child abuse [19]. Moreover, parents' and caregivers' lack of awareness and knowledge about the dangers of non-accidental injuries and their consequences may also exacerbate the problem. Poverty and unemployment, which are prevalent in the GCC, can also contribute to the incidence of child abuse as families struggle with financial difficulties and may resort to violent discipline to exert control over their children.

In the United States, each year, around more than 650,000 pediatrics are victims of abuse or maltreatment, with around 3 million annual reports received by the Child Protective Service (CPS), and two-thirds of these reports are investigated with the agency, as mentioned earlier [20]. Most of the abused children are victimized by neglect, followed by physical abuse, and then sexual abuse [20]. Nevertheless, these numbers only reflect the reported cases and not the real-life cases of abuse of all types [21]. Some institutional statistics report that the number of child abuse cases is declining; however, these statistics only represent the investigated and confirmed cases of abuse [22].

Child abuse prevention, screening, and response to the cases in the GCC region require a comprehensive approach involving different societal sectors, including healthcare providers, law enforcement agencies, schools, and social services. Healthcare providers, in particular, play a critical role in detecting and reporting cases of child abuse and providing support to affected children and their families. However, a lack of training and resources among healthcare providers may impede their ability to address cases of non-accidental injuries effectively.

Limitations

The most significant limitation of the study was the need for more sufficient and comprehensive studies, especially considering the eligibility criteria limited the region to the GCC solely. The reported cases of nonaccidental injuries in the GCC region are rarely reported, even though this does not reflect the inexistence of the abuse. There are very few studies that reported such outcomes for the analysis. Additionally, in the cases where the abuse was reported, the studies failed to define the tools used in screening and detecting the abuse. Therefore, more research is required to assess the case of nonaccidental injuries in primary pediatric care in the GCC region.

## Conclusions

In conclusion, the studies reviewed suggest that child abuse, particularly neglect, is a significant problem in the GCC region. Abusive head trauma is the most commonly associated injury, with neglect being linked to a higher incidence of internal injuries, superficial injuries, burns, ingestion, and poisoning in children. Parents were found to be the most frequent perpetrators of abuse, and cases occurred more frequently among nationals who are of lower socio-economic status. The studies highlight the need for policies and interventions to prevent child abuse and provide support to victims. It is important to note that most studies did not mention the objective assessment tools used in screening nonaccidental injuries in pediatric care in the GCC, with the ISPCAN Child Abuse Screening Tool (ICAST) being the only tool mentioned. The ICAST is a valuable tool for healthcare providers to identify cases of abuse or neglect and provide timely and appropriate interventions to prevent further harm. We recommend implementing tools for screening further refine the idea and educate societies in the GCC region regarding child abuse. Additionally, research in this area is necessary to develop effective prevention and intervention strategies.
